# Assessment of Exposure to Alcohol Vapor from Alcohol-Based Hand Rubs

**DOI:** 10.3390/ijerph9030868

**Published:** 2012-03-13

**Authors:** Vincent Bessonneau, Olivier Thomas

**Affiliations:** Environmental and Health Research Laboratory (LERES), U 1085 Institute of Research in Environmental and Occupational Health (IRSET), Advanced School of Public Health (EHESP), Avenue du Professeur Léon Bernard, 35043 Rennes, France; Email: vbessonneau@yahoo.fr

**Keywords:** inhalation exposure, alcohol-based hand rubs, passive alcoholization, healthcare workers, UV-visible spectrophotometry

## Abstract

This study assessed the inhaled dose of alcohol during hand disinfection. Experiments were conducted with two types of hand rub using two hand disinfection procedures. Air samples were collected every 10 s from the breathing zone, by bubbling through a mixture of K_2_Cr_2_O_7_ and H_2_SO_4_. The reduction of dichromate ions in the presence of alcohols was followed by UV-vis spectrophotometry. The difference in intensity of the dichromate absorption peak was used to quantify the alcohol concentration expressed in ethanol equivalent. During hygienic hand disinfection, the mean ethanol equivalent concentrations peaked at around 20–30 s for both hand rubs (14.3 ± 1.4 mg/L for hand rub 1 and 13.2 ± 0.7 mg/L for hand rub 2). During surgical hand disinfection, two peaks were found at the same time (40 and 80 s) for both hand rubs. The highest mean concentrations were 20.2 ± 0.9 mg/L for hand rub 1 and 18.1 ± 0.9 mg/L for hand rub 2. For hand rub 1, the total absorbed doses, calculated from ethanol with an inhalation flow of 24 L/min and an absorption rate of 62%, were 46.5 mg after one hygienic hand disinfection and 203.9 mg after one surgical hand disinfection. Although the use of ABHRs leads to the absorption of very low doses, sudden, repeated inhalation of high alcohol concentrations raises the question of possible adverse health effects.

## 1. Introduction

The use of alcohol-based hand rubs (ABHRs) is recommended for hand hygiene instead of antiseptic soaps owing to their antimicrobial activity against most virus and bacteria inducing healthcare associated infections [1,2]. Different types of alcohol, including ethanol, propanol and isopropanol, are used for the formulation of ABHRs. Most commercially available ABHRs contain 70% by weight of ethanol and isopropanol [3].

Nowadays, considerable effort is devoted to encouraging healthcare workers to use ABHRs to reduce hospital-acquired infections [4,5,6]. As hand hygiene compliance rates vary significantly from person to person and from one type of hospital ward to another, an overall median compliance rate of 40% has been reported in hospitals [7]. Depending on the frequency of carrying out care activities with a high risk of contamination (e.g., washing incontinent patients) and on the compliance rate, each healthcare worker may disinfect his hands on average between 5 and 30 times a day [2]. Alcohols are volatile and are easily released from gels or solutions during hand rubbing. A certain amount of alcohol may also be absorbed through the skin. Repeated exposure to alcohols could lead to passive alcoholization, possibly inducing adverse biochemical effects [3]. Despite the increasing use of ABHRs as part of hand hygiene programs, only a few studies have assessed the issue of alcohol absorption following hand disinfection [8,9,10,11,12]. The studies conducted on ethanol-based hand rubs reported that repeated hand rubbing led to blood ethanol concentrations below those known to be harmful in humans [9,10]. Below *et al.* [8] studied the dermal and pulmonary absorption of n-propanol and isopropanol during surgical and hand hygiene disinfection. As with ethanol-based hand rubs, the authors found that the amounts absorbed via inhalation and/or dermal contact were very low and probably unlikely to induce adverse health effects. Reisfield *et al.*[11,12] found that intensive use of ethanol-based sanitizer and mouthwash induces an increase in concentrations of urinary ethanol biomarkers (ethyl glucuronide and ethyl sulfate), leading to false-positive results related to ethanol consumption.

In addition to these initial studies based on theoretical considerations and blood analyses, it would be useful to determine if breathing air with alcohol vapor during hand rubbing with ABHRs, could be a health risk for healthcare workers subject to frequent exposure. So far as we are aware, no study has focused on the concentration levels of alcohols released from ABHRs into the air. This study assessed these alcohol concentration levels using a simple analytical method based on UV-vis spectrophotometry. Two hand rubs were tested, one containing ethanol only and one containing a combination of ethanol and isopropanol, during hygienic and surgical hand disinfection. 

## 2. Materials and Methods

### 2.1. Alcohol-Based Hand Rubs

Two alcohol-based hand rubs were used: hand rub 1 (Aniosgel 85 NPC, Laboratoires Anios, Lille-Hellemmes, France) contained 700 mg/g of ethanol and hand rub 2 (Germflash®) contained 560 mg/g of ethanol and 90 mg/g of isopropanol. The hand rubs contained agents for skin protection but no perfume or coloring.

### 2.2. Hand Rubbing Procedures

According to the manufacturers recommendations [13], the hygienic hand disinfection procedure consists in applying 3 mL of gel (2.8 ± 0.03 g, *n* = 5) to the palm of one hand and rubbing the hands together for 30 s until the solution has completely evaporated. For surgical hand disinfection, 3 mL of gel (2.8 ± 0.03 g, *n* = 5) was applied to the hands and rubbed over the hands and forearms for 45 s. This was carried out twice for a total of 90 s. All experiments were carried out by the same person.

### 2.3. Experimental Apparatus

**Figure 1:**
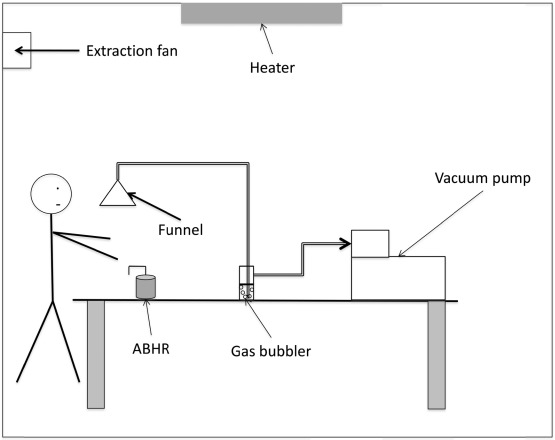
Experimental setup.

Figure 1 shows the experimental setup used. Experiments were conducted in a room with a volume of 55 m^3^. The room temperature was maintained at 21 ± 1 °C by a heater. The relative humidity was not controlled but was measured during each experiment. An extractor fan provided a constant air exchange rate (AER) of 12 ± 1 h^−1^. The AER was checked by monitoring the decrease of a tracer gas, SF_6_, using an Innova 1412 photoacoustic gas analyzer (LumaSense Technologies A/S, Ballerup, Denmark).

Alcohol vapor released during hand disinfection was sampled from the breathing zone (150 cm), passed through a glass funnel (10 cm ID) and silicone rubber tube (2 cm ID) and bubbled through 50 mL of a solution of K_2_Cr_2_O_7_ (8.33 mmol/L) in concentrated H_2_SO_4_ (18 mol/L) at a flow rate of 1.4 L/min using a vacuum pump (Bioblock Scientific). A cylindrical glass gas bubbler (3 cm ID × 20 cm high) was used as a reactor. Before each experiment the solution was freshly prepared by combining 8.3 mL of K_2_Cr_2_O_7_ (8.33 mmol/L) with 25 mL of H_2_SO_4_ (18 mol/L) and 16.7 mL of ultra-pure water in a 50 mL flask. The alcohol was absorbed through the solution and assayed using the following redox reaction:


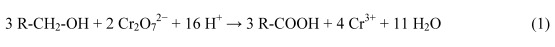


where R is CH_3_ or C_2_H_5_ for ethanol and isopropanol respectively.

This is the same reaction as that used in the color change breath alcohol test. The exposure was assessed by collecting five 10 s air samples every 10 s for 50 s for hygienic hand disinfection and 110 s for surgical hand disinfection. Blank samples were collected before each experiment to ensure there were no other chemicals that might react with the reagents. Owing to the high concentration of acid, the alcohol reacted instantaneously with K_2_Cr_2_O_7_, and there was no passthrough, the alcohol was entirely collected by absorption and reaction into a single gas bubbler at this flow rate. After each experiment, the sampling flow rate was checked using a bubble flow meter Gilian Gilibrator 2 (Sensidyne, Clearwater, FL, USA). When the flow rate varied by more than 5%, the sample was rejected and the experiment was repeated. 

### 2.4. Analysis of Alcohol

The reduction of hexavalent chromium (Cr (VI)) into trivalent chromium (Cr (III)) by alcohols was measured by UV-vis spectrophotometry, with a strong absorption peak around 440 nm. This method was first proposed for the spectrophotometric determination of chemical oxygen demand (COD) in water [14]. The absorption spectrum of each sample was recorded using a Perkin Elmer Lambda 25 spectrophotometer (Perkin Elmer) for 3.5 mL samples in a 10 mm thick flow-through quartz cell. The spectra were measured from 400 to 550 nm at 1 nm steps with a scan speed of 8 nm/s.

**Figure 2:**
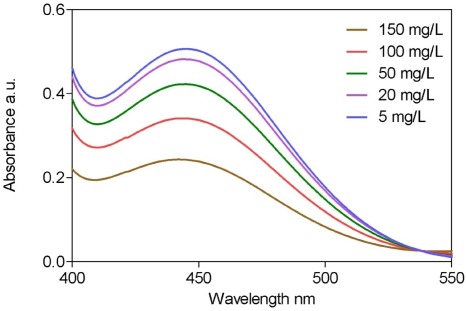
UV-vis absorption spectra measured from 400 to 550 nm for ethyl alcohol concentrations in mixture ranged from 5 to 150 mg/L.

Solutions of K_2_Cr_2_O_7_ (8.33 mmol/L) with 25 mL of H_2_SO_4_ (18 mol/L) containing 4 mL of ethanol at five concentration levels in the range 5–150 mg/L were analyzed for calibration. Figure 2 shows the spectra obtained from the multipoint calibration. Quantification was performed by measuring the dichromate absorption peak at 440 nm. Table 1 presents the characteristics of the method.

**Table 1:** Characteristics of the UV-vis spectroscopic method for the determination of alcohols.

Parameter	Value
Wavelength range(nm)	400–550
Wavelength quantification (nm)	440
Concentration range (mg/L)	5–150
Determination coefficient R²	0.998
Sensitivity (mg/L)	0.4
Limit of detection (mg/L)	5
Relative standard deviation (%) (n = 5)	3

After analysis, all mixtures were collected in a dedicated tank, which was removed by a waste disposal firm, owing to the toxicity of hexavalent chromium. 

### 2.5. Data Analysis

Statistical analyses were performed using GraphPad Prism version 5.01 for Windows (GraphPad Software, San Diego, CA, USA). For each time point, the arithmetic mean and the standard deviation of the ethanol equivalent concentration in air was calculated. The non-parametric Mann-Withney test was applied to determine whether the median concentrations measured from gel 1 and gel 2 differed significantly. A significance level of 0.05 was used for all tests.

## 3. Results

### 3.1. Hygienic Hand Disinfection

**Figure 3:**
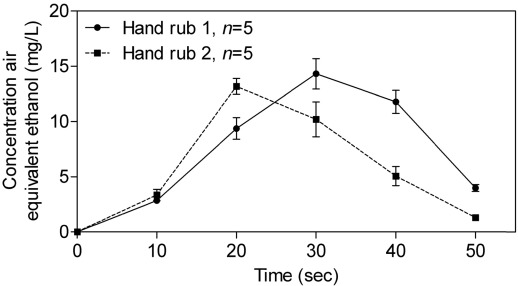
Evolution of alcohols concentrations in air during hygienic hand disinfection (arithmetic mean ± SD) with hand rub 1 and hand rub 2.

Figure 3 shows the alcohol concentration measured in air during hygienic hand disinfection with hand rub 1 and hand rub 2. The mean ethanol concentration in air increased during hand disinfection and peaked after 30 s with hand rub 1 and after 20 s with hand rub 2. The highest mean concentrations measured with hand rub 1 (14.3 ± 1.4 mg/L) and with hand rub 2 (13.2 ± 0.7 mg/L) were not statistically different (*p* = 0.23). There was no significant difference (*p* = 0.09) in mean concentration at 10 s between hand rub 1 (2.9 ± 0.2 mg/L) and hand rub 2 (3.3 ± 0.5 mg/L). For both hand rubs, the mean concentrations in air decreased rapidly and returned to zero 20 s after the end of hand disinfection (after 50 s).

### 3.2. Surgical Hand Disinfection

**Figure 4:**
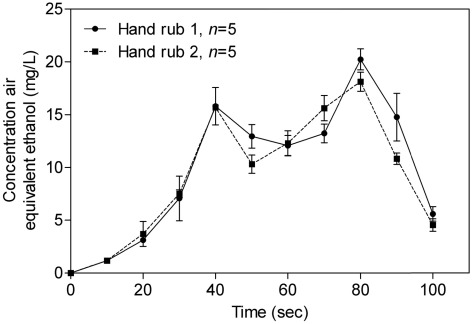
Evolution of alcohols concentrations in air during surgical hand disinfection (arithmetic mean ± SD) with hand rub 1 and hand rub 2.

The mean alcohol concentration in air for hand rub 1 and hand rub 2 is shown in Figure 4. For both hand rubs, the mean alcohol concentration gradually increased and reached a first peak at 40 s. 

After the beginning of the second rubbing, the mean alcohol concentration increased with hand rub 2, whereas the mean alcohol concentration with the hand rub 1 continued to decrease. The highest mean concentration was found at 80 s with hand rub 1 (20.2 ± 0.9 mg/L) and with hand rub 2 (18.1 ± 0.9 mg/L) and there was no statistical difference (*p* = 0.1). Before the end of the second hand disinfection, the mean alcohol concentration decreased significantly for both hand rubs. 

### 3.3. Exposure Assessment

The results from the experiments conducted with hand rub 2 were not used to assess the exposure via inhalation. Hand rub 2 contained isopropanol which, unlike ethanol, is known to be absorbed easily through the skin (dermal permeability coefficient of 1350 cm/h) [15,16]. Exposure to ethanol during both hygienic and surgical hand disinfection procedures was estimated by combining the alcohol concentration measured, the breathing frequency and the tidal volume. Assuming that healthcare workers perform light physical activities during their work-shift, the breathing frequency was calculated from the average inhalation rate of 24 L/min defined by the U.S. EPA [17] and a tidal volume of 0.5 l. This gave a breathing frequency of 48 breaths/min and a breathing cycle of 0.6 s inhalation, 0.6 s exhalation and 0.25 s break. The total inhaled dose after hand disinfection was calculated by calculating the sum of the ethanol inhaled on each breathing cycle:


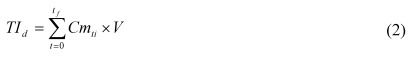


where TI_d_ is the total inhaled dose (mg), Cm_ti_ is the average concentration of ethanol (mg/L) for inhalation at time ti and V is the tidal volume (0.5 L). Cm_ti_ can be calculated using Equation (3):


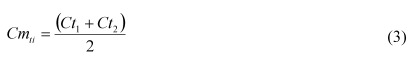


where Ct_1_ and Ct_2_ are the concentrations of ethanol at the beginning and end of inhalation between t_1_ and t_2_, respectively, assuming that the concentration variation is linear between t_1_ and t_2_. This assumption of linearity was extended to the concentrations between the measurements made every 10 s (Figure 3 and Figure 4).

**Figure 5:**
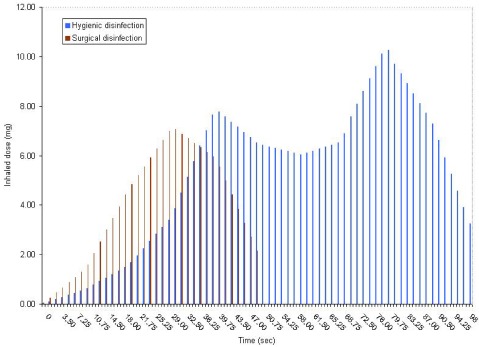
Evolution of inhaled doses (mean) during hygienic and and surgical hand disinfection with hand rub 1.

Figure 5 presents the variation of inhaled doses of ethanol during hygienic and surgical hand disinfection with hand rub 1, taking account of the breathing cycle. During the 30 s hygienic hand disinfection, the inhaled dose of ethanol ranged from 0.04 to 7.06 mg, giving a cumulative exposure of 74.9 mg. After 90 s surgical hand disinfection, the total inhaled dose of ethanol was 328.9 mg. The highest doses were found after 40 s (7.8 mg) for hygienic hand disinfection and after 80 s (10.3 mg) for surgical hand disinfection.

Based on an absorption efficiency through the lungs of 62% [18], the estimated absorbed dose was 46.5 mg after one hygienic hand disinfection and 203.9 mg after one surgical hand disinfection. Based on the composition of the hand rub 1 and the quantity of the solution applied, users were exposed to 1.96 g of ethanol alcohol during hygienic hand disinfection and 3.92 g during surgical hand disinfection. The ratio between the absorbed dose and the quantity applied for each hand rubbing gives the rate of ethanol absorption which was 2.4% for hygienic hand disinfection and 5.2 % for surgical hand disinfection.

## 4. Discussion

So far, as we are aware, no previous study has focused on alcohol concentration in inhaled air resulting from the use of ABHRs. Most of the studies covering exposure to alcohols during hand disinfection have measured blood alcohol concentrations. Consequently, this study extends knowledge on alcohol vapor concentrations during hand rubbing using a simple analytical method based on UV-vis spectrophotometry. The hand rubs were selected in order to study the concentration of alcohols released into the air from the two main categories of commercially available ABHRs: ethanol and combined alcohols [3].

### 4.1. Experimental Conditions

The main limitation of the study was the non-specificity of the UV-vis spectrophotometric method. For experiments conducted with hand rub 2, the contribution of isopropanol to the total alcohol levels measured could not be estimated. Many of the volatile organic compounds (VOCs) frequently measured in indoor air can easily react with the solution of K_2_Cr_2_O_7_ in strong acid conditions. However, the experimental conditions were designed to ensure that oxidizing compounds needed to reach a concentration in air as high as 0.9 mg/L to have a measurable decrease in the dichromate absorption peak (440 nm). Before each experiment, a blank sample was collected to ensure that no other oxidizable compound was present in the room. The method also used a highly concentrated mixture of K_2_Cr_2_O_7_ and H_2_SO_4_ that required strict compliance with safety rules. Given the potential risk of this technique, other analytical methods for measuring alcohols in air, avoiding the use of dangerous chemicals, might be preferred [19,20]. 

It is also important to note that the concentrations values presented in Figure 3 and Figure 4 were the average concentrations measured during the sampling period (*i.e.*, 10 seconds) and did not correspond to the actual concentrations at 10 seconds. Consequently, the concentrations reported were probably lesser than the actual concentrations. 

In our study, the glass funnel was placed near the breathing zone of the subject, in order to be representative of the distance between the hands and the respiratory tract. The subject kept the same distance, as far as possible, between the hands and the funnel for all experiments. A quite short distance between the hands and the funnel probably increased the amount of alcohols sampled and lead to over estimation of the associated absorbed dose.

### 4.2. Comparison of Hand Rub 1 and Hand Rub 2

Alcohol was not released at the same rate during hygienic hand disinfection. As hand rub 2 contained less alcohol (65% by weight) than hand rub 1 (70% by weight), the peak concentration was sooner from hand rub 2 (peak at 20 s) than from hand rub 1 (peak at 30 s). High standard deviations were found for high concentrations, probably due to the difficulty of ensuring the same hand rubbing intensity in each experiment.

Surprisingly, alcohol seemed to be released at the same rate (peaking at 40 s) during the first part of the surgical hand disinfection (0–40 s). Although, during the second rubbing (50–100 s), the mean alcohol concentrations peaked at the same time (80 s), the alcohol was released at different rates, depending on the amount of alcohol contained in the hand rubs.

### 4.3. Risk Assessment

Absorption of high doses of ethanol is known to have adverse effects on many organs. Most studies have concerned the consumption of alcoholic beverages both for acute (binge drinking) and chronic consumption [21]. Several literature reviews have shown that an increased health risk is unlikely after occupational exposure to ethanol through inhalation and dermal contact [22,23,24]. A few studies have assessed the absorption of ethanol during hand disinfection [9,10]. Both of these reported that the blood ethanol concentration was below the toxic level for humans (50 mg/L) after successive application of hand rubs containing various proportions of ethanol (from 55% to 95% by weight).

The results of this study suggest that the total amount of ethanol absorbed after a 30 s hygienic hand disinfection was 46.5 mg and after a 90 s surgical hand disinfection was 203.9 mg. These results are slightly higher than those reported by Kramer *et al*. [9] for the concentration in blood. They reported ethanol absorption of 31.5 mg (630 mg after 20 hygienic hand disinfections) and 154.2 mg (1542 mg after 10 surgical hand disinfections), with a hand rub containing 85 mg/g of ethanol. These discrepancies are probably due to the assumptions regarding the inhalation rate (24 L/min) and the efficiency of pulmonary absorption (62%). The average inhalation rate for adult males performing light physical activities such as walking [17] used probably overestimates the real respiration of healthcare workers during working hours. Various factors such as the inhalation rate and the tidal volume (or even holding the breath while rubbing) could affect the inhalation of ethanol, leading to an absorption efficiency ranging from 30% to 80% [23,25]. Based on the maximum of 30 hand disinfections reported per working day [2], the corresponding absorbed dose would be around 950 mg, approximately one tenth the dose of ethanol absorbed after one glass of wine (9.6 g).

Although the calculated absorbed dose during the working day was very low, health workers would frequently be exposed, for short periods, to high concentrations of ethanol during hand disinfection. The 15 min short-term exposure level or STEL (9.5 mg/L) adopted in France was exceeded for 25 s of hygienic hand disinfection (from 20 to 45 s) and for 63 s (from 32 to 95 s) of surgical hand disinfection. The requirement for repeated hand disinfection may lead to a cumulated exposure of more than 10 min in a working day where the STEL is exceeded. This poses the question of possible health effects following sudden and repeated inhalations of high concentration of ethanol. The report of the Health Council of the Netherlands [23] on the exposure of workers to ethanol indicated that a sudden change in ethanol concentrations from 0 to 3.6 mg/L may cause temporary irritation. Furthermore, a concentration of 17 mg/L or more was reported as unbearable for unacclimatized persons. A few studies have assessed the effects of inhalation of alcohol vapor on self-administration of alcohol in Wistar rats [26,27]. O’Dell *et al*. [27] showed that intermittent exposure to alcohol vapor significantly increased self-administration of alcohol compared to continuous exposure. Gilpin *et al*. [26] found that brain alcohol concentrations in alcohol-experienced rats decreased faster than for alcohol-naïve rats after intermittent exposure. However, these findings cannot be extrapolated to inhalation of alcohol during intensive hand rubbing, owing to obvious experimental differences. Gilpin *et al*. [26] and O’Dell *et al*. [27] exposed Wistar rats (weight ranging from 180 to 350 g) for 14 h, against an exposure of 50 s or 100 s in this assessment of the exposure of healthcare workers.

## 5. Conclusions

This study provides experimental data using a simple method to improve knowledge about alcohol inhalation during hand disinfection. Hand rubbing is a necessary professional practice for reducing healthcare associated infections and these first results show that unintentional absorption of alcohol by healthcare workers may have different effects depending on the time scale. On the one hand, the calculated absorbed dose from hand disinfection for a whole working day of a nurse disinfecting the hands 30 times was lower than the dose known to be harmful on consumption of alcoholic beverages. This dose is approximately one tenth the alcohol content of a glass of wine and so there is no evidence for considering that the unintentional absorption of alcohol from ABHRs poses a health threat. However, special attention should be paid to pregnant healthcare workers, for which exposure to alcohol, even at low doses, may induce possible harmful effects on the fetus. On the other hand, this study showed that the 15 min STEL may be exceeded for a few tens of seconds during hand disinfection. Little is known about health effects of sudden, repeated exposure to high concentrations of alcohol. As the use of ABHRs will increase owing to its indisputable effectiveness in reducing healthcare associated infections, protection at the point of use must be provided if it is shown that sudden, repeated exposure to high concentrations of alcohol leads to adverse health effects. Further studies are required.
